# Imbalance of T cell subsets: a core event that mediates the progression of T2DM and its complications

**DOI:** 10.3389/fimmu.2025.1688392

**Published:** 2025-11-21

**Authors:** Xiang Xie, Fan Li, Qian Wu, Chunlan Zeng, Xi Chen, Wenwen Wang, Chunxiang Zhang, Huan Chen

**Affiliations:** 1 School of Basic Medical Science, Southwest Medical University, Luzhou, China; 2 Public Center of Experimental Technology, Southwest Medical University, Luzhou, China; 3 Key Laboratory of Medical Electrophysiology, Southwest Medical University, Luzhou, China; 4 Ministry of Education & Medical Electrophysiological Key Laboratory of Sichuan Province, Southwest Medical University, Luzhou, China; 5 Nucleic Acid Medicine of Luzhou Key Laboratory, Southwest Medical University, Luzhou, China

**Keywords:** T cell subset imbalance, T2DM, insulin resistance, inflammation, T2DM complications

## Abstract

Type 2 diabetes mellitus poses a substantial global health burden, increasing evidence highlights the critical role of T cells in promoting T2DM progression. This review provides an overview of the mechanisms by which specific T cell subsets drive T2DM pathogenesis and its complications, while also highlighting emerging immunotherapeutic strategies. Preceding overt T2DM, T cells infiltrate insulin-sensitive tissues early, and a skewing of T cell subsets toward pro-inflammatory phenotypes leads to an imbalance that fosters inflammation and M1 macrophage polarization, driving the development of T2DM. In addition, this T cell subset imbalance contributes to disease progression by inducing insulin resistance and β-cell dysfunction. As T2DM progresses, the T cell subset imbalance and their tissue infiltration extend to the cardiovascular system, kidneys, retina, brain, and peripheral tissues—contributing to diabetic complications such as atherosclerosis, diabetic kidney disease, diabetic retinopathy, Alzheimer’s disease, and diabetic foot ulcers. The evidence summarized in this review underscores the central role of T cell subset imbalance in the progression of T2DM and its associated complications. Building on these findings, we also examine both established and emerging therapeutic strategies, including restoring T cell subset balance, modulating T cell–derived pro- and anti-inflammatory cytokines, and shifting macrophage polarization driven by pro-inflammatory T cells, to offer critical insights for future clinical intervention. T cell subset imbalance is a core driver of the progression of T2DM and its complications, and targeting T cell dysregulation represents a promising frontier in T2DM therapy.

## 1 Introduction

Diabetes, a complex and multifaceted disease, poses significant global health challenges. As reported by the International Diabetes Federation, approximately 537 million adults aged between 20 and 79 are living with diabetes worldwide as of 2021 (1). Alarmingly, this number is forecasted to rise to 643 million by 2030 and 783 million by 2045 (1). Type 2 diabetes mellitus (T2DM) accounts for nearly 90% of all cases and is primarily characterized by insulin resistance, chronic low-grade inflammation, and progressive β-cell dysfunction. Recent advancements in understanding the pathological mechanisms of T2DM potentially offer novel prevention strategies and innovative treatment options to better control its escalating prevalence (2, 3).

Increasing evidence has highlighted the pivotal role of the immune system in the development and progression of T2DM (4). In the early stages of T2DM, there are multiple types of immune cells infiltrating in insulin-sensitive tissues, where they disrupt tissue homeostasis, exacerbate insulin resistance, and perpetuate systemic inflammation (5–9). Among these, T cells are increasingly appreciated as key orchestrators of inflammatory responses in T2DM (4, 10). Notably, T cell infiltration into visceral adipose tissue occurs early in disease development—recruiting macrophages and neutrophils—and plays a critical role in initiating and sustaining the inflammatory milieu (9, 11, 12).

An imbalance between pro-inflammatory and anti-inflammatory T cell subsets, both systemically and within specific tissues, is a central feature of T2DM-associated immune dysregulation (13). This imbalance is shown by an increased prevalence of pro-inflammatory T cells—including CD8^+^ cytotoxic T lymphocytes, T helper 1 (Th1), Th17, and Th22 subsets—and a corresponding decrease in anti-inflammatory subsets such as regulatory T cells (Tregs) and Th2 cells (10, 14, 15). These shifts promote a chronic low-grade inflammatory milieu that exacerbates insulin resistance and β-cell dysfunction (10, 14, 15). Systemic immune dysregulation alone cannot fully explain the tissue-specific damage observed in diabetic complications. As T2DM advances, tissue-specific infiltration of pro-inflammatory T cells becomes increasingly evident in metabolically and vascularly vulnerable organs, including the vasculature, kidneys, and retina. Within these tissues, infiltrating T cells actively contribute to local inflammatory cascades—promoting endothelial dysfunction, fibrosis, and microvascular injury—that drive the progression of diabetes-related complications. In atherosclerosis (AS), T2DM is associated with increased infiltration of pro-inflammatory T cell subsets, particularly Th1 and Th17 cells, into atherosclerotic plaques. These cells promote the recruitment of additional immune cells, induce vascular cell apoptosis, and compromise the structural integrity of plaques, thereby increasing the risk of rupture and thrombosis (16, 17). In diabetic kidney disease (DKD), a hallmark feature is the accumulation of immune cells, particularly CD4^+^ T cells, within renal tissues. These T cells promote glomerular damage, podocyte injury, and interstitial fibrosis through the release of pro-inflammatory cytokines and direct cellular injury mechanisms (18, 19). Similarly, in diabetic retinopathy (DR), pro-inflammatory T cell infiltration damages the retinal microvasculature, promoting blood-retina barrier disruption, vascular leakage, and pathological neovascularization, all of which contribute to the progression of retinal damage (20, 21).

Despite growing interest in T cell involvement in T2DM, critical knowledge gaps remain. In particular, the precise contributions of distinct T cell subsets to the onset and progression of T2DM are not fully elucidated. Furthermore, the molecular and cellular mechanisms linking T cell imbalance to tissue-specific pathology warrant deeper investigation. Understanding how T cell imbalance contributes to tissue-specific pathology is essential for identifying targeted immunomodulatory therapies. This review discusses the engagement of T cells in the progression of T2DM, emphasizing how T cell subset imbalance drives the development of T2DM by initiating inflammation, interacting with other immune cells, directly impairing insulin sensitivity, and promoting β-cell dysfunction. Moreover, we extend our discussion to the roles of T cell subset imbalance in T2DM-associated complications, such as AS, DKD, and DR. Finally, we discuss current and emerging therapeutic strategies aimed at correcting T cell subset imbalance in diabetes and its complications. With its breakthrough focus on elucidating the pivotal role of T cell subset imbalance in T2DM and its complications, this review pioneers the discussion of novel immunotherapies and their clinical promise. It thus bridges basic mechanisms and clinical application, providing a highly potential therapeutic approach for T2DM.

## 2 T cells initiate infiltration during the early development of T2DM

**Figure 1:**
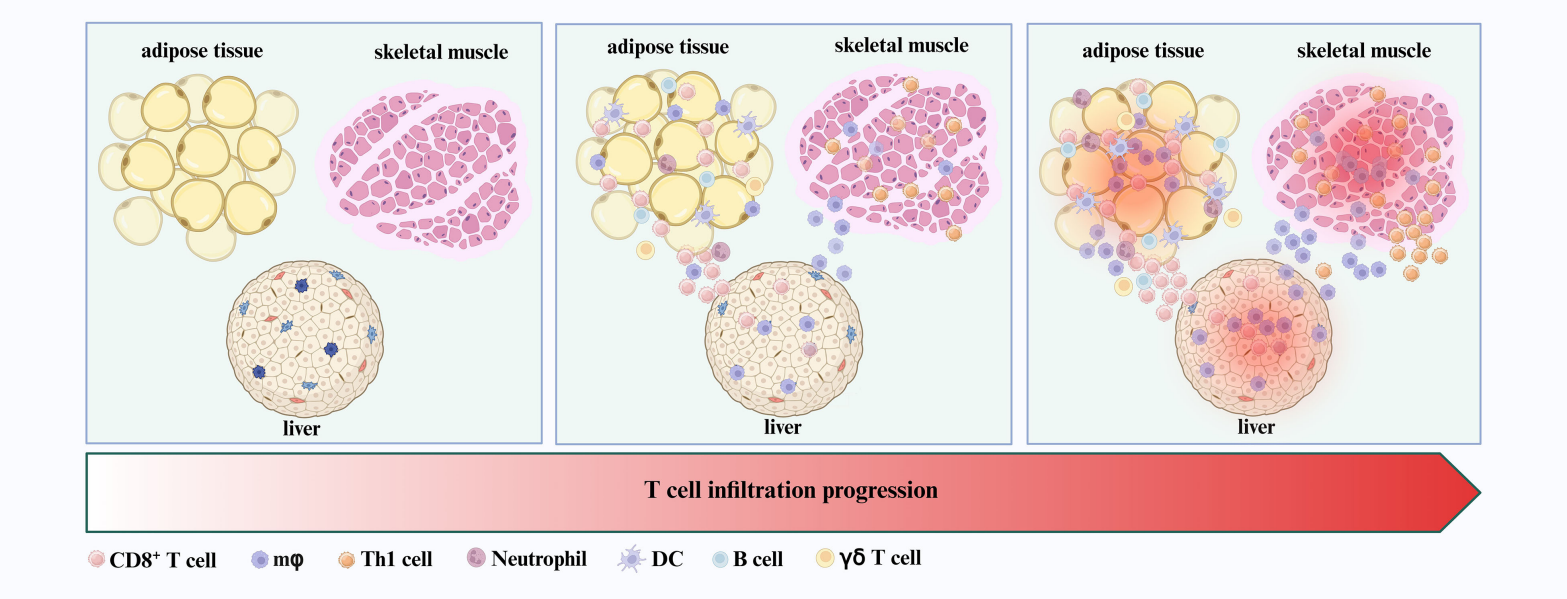
T cells as early infiltrators in insulin-responsive tissues. CD8^+^ T cells and Th1 cells, along with macrophages, neutrophils, DCs, B cells, and γδ T cells, are among the first immune populations to infiltrate insulin-responsive tissues—including adipose tissue, skeletal muscle, and liver—during the initial stages of T2DM development.

A wide range of immune cells, including macrophages, neutrophils, dendritic cells (DCs), B cells, and particularly T lymphocytes, infiltrate metabolic tissues such as adipose tissue, skeletal muscle, and liver (5–9). Among them, macrophages play a direct role in local and systemic insulin resistance and inflammation, significantly driving the development of T2DM (5, 22). Notably, reports indicate that T cells are among the first immune cells to infiltrate adipose tissue, preceding and facilitating macrophage infiltration during the early stages of T2DM development (9, 23) (**Figure 1**). For example, Kintscher et al. documented a notable decline in insulin sensitivity and a surge in T-cell infiltration within visceral adipose tissue in mice after 5 weeks of high fat diet (HFD) (23, 24). Macrophage infiltration was not observed after 5 weeks of HFD and only appeared around 10 weeks of HFD (23). These findings suggest that T cell infiltration into adipose tissue predates macrophage recruitment during the development of insulin resistance. Moreover, Nishimura et al. demonstrated that CD8^+^ cytotoxic T cell infiltration took precedence over macrophage accumulation in adipose tissue (9). Interestingly, when CD8^+^ T cells were introduced to lean adipose tissue, there was an induction of macrophage differentiation and a local inflammatory cascade (9). Subsequent experiments revealed that treating with a CD8-specific antibody considerably curtailed macrophage accumulation, thereby mitigating adipose inflammation and insulin resistance in HFD mice (9). Additionally, the γδ T cells also play early roles in orchestrating adipose inflammation. γδ T cells have been found to increase in adipose tissue during the early phase of HFD feeding and have been shown to promote macrophage accumulation and inflammatory responses (24).

In addition to macrophages, other immune cells are also rapidly recruited to adipose tissue and interact with T cells to exacerbate inflammation. For example, neutrophils infiltrate adipose tissue early in obesity, promoting inflammation through the release of neutrophil extracellular traps (NETs) (6). T cell-derived cytokines—particularly interleukin 17 (IL-17) 17 from Th17 cells and interferon-γ (IFN-γ) from Th1 cells—can enhance neutrophil recruitment, leading to increased production of reactive oxygen species (ROS) and further NETs formation. These processes exacerbate tissue damage and inflammation (6, 11, 12, 25). DCs also accumulate in adipose tissue in db/db mice and exhibit enhanced capacity to promote Th1 and Th17 differentiation in both mice and humans (7, 26). IFN-γ produced by Th1 cells further enhances DC antigen-presenting activity by upregulating major histocompatibility complex (MHC) class II expression, thereby sustaining local inflammation (27, 28). B cells have been reported to accumulate early in the adipose tissue of obese mice and contribute to inflammation through the production of pathogenic IgG and pro-inflammatory cytokines (8). Notably, the interaction between CD40 ligand (CD40L) expressed on T cells and CD40 on B cells provides a critical co-stimulatory signal, promoting B cell proliferation, differentiation, and antibody production (29). This T–B cell crosstalk further amplifies the inflammatory network within adipose tissue.

Beyond adipose tissue, T cell infiltration also occurs early in other insulin-responsive organs, such as skeletal muscle and liver. Skeletal muscle is a primary site of glucose uptake and a key target of insulin signaling. In obese mice, αβ T cells—including Th1 subsets—accumulate in skeletal muscle during the early stages of insulin resistance (30, 31) (**Figure 1**). The deletion of αβ T cells not only shields mice from HFD-induced hyperglycemia and insulin resistance, but also suppresses macrophage infiltration and reduces inflammatory cytokine expression in both skeletal muscle (30). Furthermore, the adoptive transfer of Th1 cells into the HFD-fed mice with deletion of αβ T cells precipitated enhanced macrophage infiltration and inflammation in skeletal muscle (30). Similarly, an early surge in CD8^+^ T cells infiltration is noted in the livers from both obese humans and mice (32, 33) (**Figure 1**). The depletion of CD8^+^ T cells via anti-CD8a antibody treatment ameliorates hepatic inflammation in obese mice by reducing the abundance of both resident and infiltrating macrophages within the liver (32, 33).

In summary, T cells are among the earliest immune cells to infiltrate metabolically active tissues during the initial stages of T2DM pathogenesis. Their early recruitment not only precedes but also promotes the subsequent infiltration and activation of other immune cell populations—including macrophages, neutrophils, DCs, and B cells—thereby establishing a sustained pro-inflammatory microenvironment (**Figure 1**).

## 3 T cell subset imbalance promotes the development of T2DM

Clinical studies have demonstrated that individuals with obesity and T2DM exhibit an elevated level of total T cells in peripheral blood, accompanied by a marked reduction in circulating Tregs. Furthermore, the ratios of Tregs to Th1 and Tregs to Th17 cells are significantly diminished (13, 34). These alterations reflect a systemic immune imbalance characterized by a disruption in T cell subset homeostasis under hyperglycemic conditions. This perturbed T cell subset balance fosters a permissive environment for the accumulation and activation of tissue-specific T cells, thereby driving the key pathogenic features of T2DM: chronic inflammation and insulin resistance (10, 14). Furthermore, this immune imbalance directly contributes to pancreatic islet dysfunction, further exacerbating disease progression by impairing insulin secretion and β-cell survival (35, 36) (**Table 1**).

**Table 1:** T cell subset imbalance promotes the development of T2DM.

T cell subset	Cytokine	Affected tissue	Pathological outcome
CD8^+^ T cells	TNF-α, IFN-γ, granzyme B	adipose tissue, liver, islet	inflammation, insulin resistance, islet β-cell apoptosis and dysfunction
Th1 cells	IFN-γ	skeletal muscle, islet	inflammation, insulin resistance, islet β-cell apoptosis and dysfunction
Th17 cells	IL-17, IL-21, IL-22, IL-17 F, GM-CSF	islet	inflammation, insulin resistance
Th22 cells	IL-22	adipose tissue, liver, islet	inflammation, insulin resistance
Tregs	IL-10, TGF-β	adipose tissue, skeletal muscle, islet	inhibit inflammation
Th2 cells	IL-4, IL-5, IL-10	adipose tissue, skeletal muscle, islet	inhibit inflammation
γδ T cells	–	adipose tissue	inflammation
iNKT cells	–	adipose tissue	ameliorate insulin resistance, reduce blood glucose

### 3.1 T cell subset imbalance initiates inflammation

CD8^+^ T cells play a central role in triggering and amplifying inflammatory processes. In T2DM, there is a notable increase in CD8^+^ T cells both in circulation and within insulin-sensitive tissues, such as skeletal muscle, liver, and, most notably, adipose tissue (9, 23). This phenomenon has been consistently observed in both human studies and HFD-induced obese mouse models, where CD8^+^ T cells infiltrate adipose tissue early during the onset of obesity and insulin resistance (9). Once recruited, CD8^+^ T cells secrete pro-inflammatory cytokines, including IFN-γ and tumor necrosis factor-α (TNF-α), exacerbating inflammation (9). Additionally, these cells actively facilitate the recruitment of other immune cells, such as macrophages, monocytes, and additional T cells, establishing a self-perpetuating inflammatory loop (9, 23, 37). Notably, depletion of CD8^+^ T cells in HFD-induced obese mice leads to a significant reduction in pro-inflammatory cytokine levels and overall inflammation (9).

**Figure 2:**
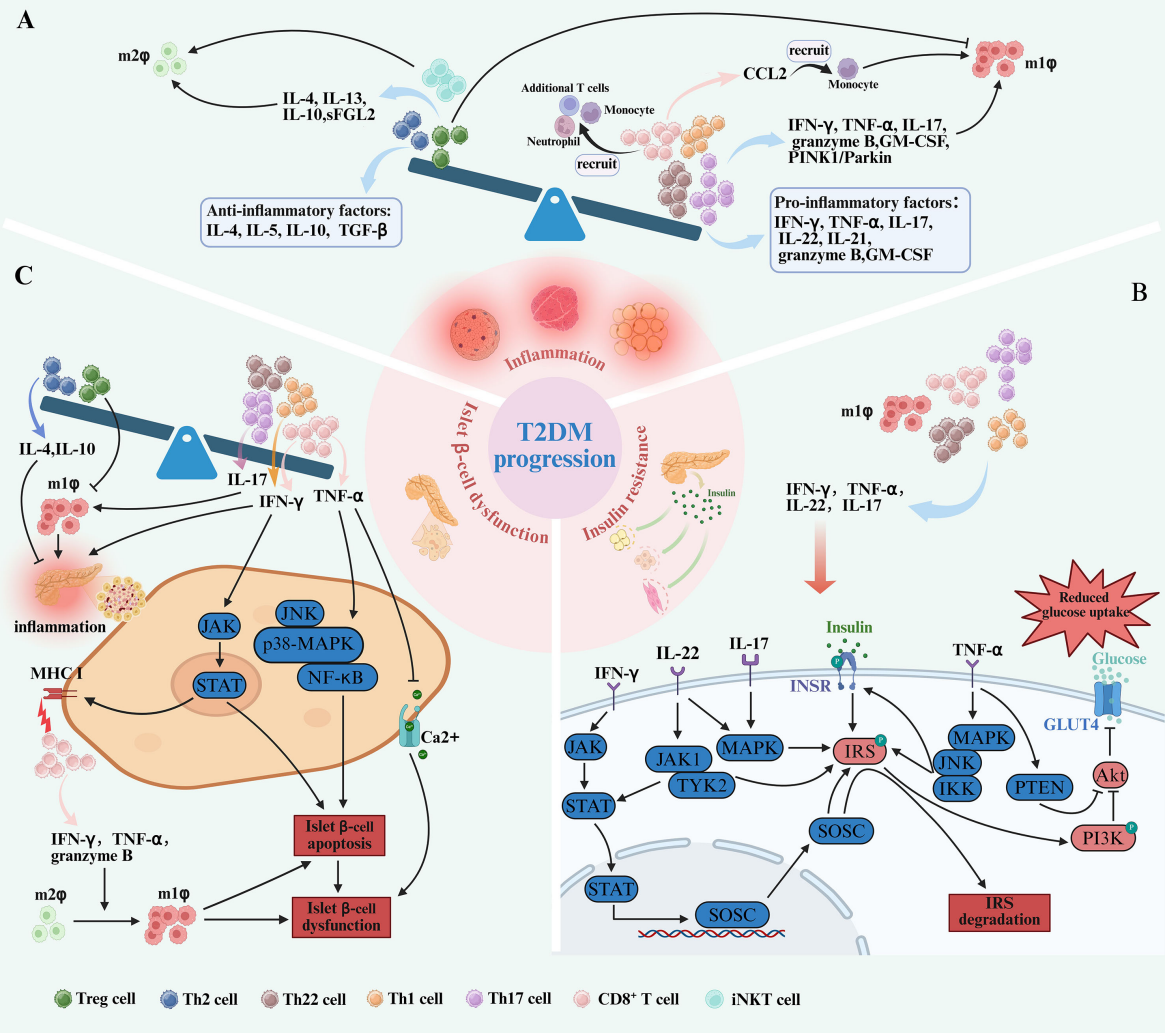
T cell subset imbalance contribute to the pathogenesis of T2DM. **(A)** In the progression of T2DM, the imbalance of T cell subsets is caused by the increase of pro-inflammatory T cells, including CD8^+^ T cells, Th17, Th22, and Th1 cells and the decrease of anti-inflammatory T cells, including Tregs and Th2 cells. This is accompanied by elevated levels of cytokines pro-inflammatory T cells secrete, such as IFN-γ, TNF-α, GM-CSF, granzyme B, IL-21, IL-22and IL-17. Conversely, anti-inflammatory subsets are reduced, leading to decreased secretion of cytokines including IL-5, IL-4, IL-10, and TGF-β. Both IFN-γ, TNF-α, IL-17, IL-22 and IL-21 secreted by pro-inflammatory T cells and CD8^+^ T cells recruiting monocytes through the release of CCL2 promote pro-inflammatory M1 macrophage polarization. In contrast, M2 macrophage polarization, which depends on cytokines like IL-10, IL-4, IL-13, sFGL2 and iNKT cells, is diminished. The interaction between T cells and macrophages establishes a pro-inflammatory loop, driving the progression of T2DM. **(B)** Pro-inflammatory T cell subsets—Th17 cells, Th22 cells, CD8^+^ T cells, and Th1 cells—can directly impair insulin sensitivity through the secretion of IL-17, IL-22, TNF-α, and IFN-γ. IFN-γ activates the JAK-STAT signaling pathway, promoting SOCSs transcription. SOCSs disrupts insulin signaling by targeting IRS-1 for phosphorylation and degradation, thereby impairing PI3K/Akt pathway, downregulating GLUT4 and reduce glucose uptake. TNF-α activates MAPK, JNK, and IKK, which results in phosphorylation of the IR and IRS-1, leading to insulin resistance on glucose uptake and GLUT4 translocation. Similarly, IL-17 and IL-22 activate the MAPK pathway, causing IRS-1 phosphorylation and further impairing insulin signaling. In addition, IL-22 induces the activation of JAK1 and TYK2, promoting the translocation of STAT3 into the nucleus, impairing insulin sensitivity. **(C)** An increase of CD8^+^ cells, Th17 cells and Th1 cells also plays a role in pancreatic β-cell dysfunction by activating the JAK-STAT, JNK, p38-MAPK, NF-κB signaling pathways and promote islet inflammation through cytokines such as IFN-γ, and TNF-α. Furthermore, IFN-γ enhances the expression of MHC-I on β-cells by activating the JAK-STAT, making them more susceptible to attack by cytotoxic CD8^+^ T cells. CD8^+^ T cells secrete granzyme B, TNF-α and IFN-γ, both of which promote M1 polarization and contribute to β-cell dysfunction. In addition, the number of Th22 cells also increased. These shifts, along with decreased Tregs and Th2 cells, create a pro-inflammatory microenvironment that promotes islet inflammation.

Th1 cells, characterized by their production of IFN-γ, represent another critical pro-inflammatory subset in T2DM (38). Compared with healthy control subjects, obese individuals harbor increased numbers of activated circulating Th1 cells (39). These cells preferentially infiltrate insulin-sensitive tissues, including adipose tissue, skeletal muscle, and the liver, where they not only sustain inflammation but also exacerbate it through cross-talk with resident macrophages (40, 41). This interaction is mediated by the secretion of IFN-γ, TNF-α, and IL-2 (41, 42) (**Figure 2A**). Consequently, this signaling cascade leads to an increased production of inflammatory cytokines and adipokines, further amplifying the inflammatory milieu. Notably, depleting Th1 cells in HFD obese mice has been shown to ameliorate inflammation and reduce the influx of macrophages (43).

Similarly, Th17 cells also play a distinct role in T2DM-related inflammation (44). Elevated levels of Th17 cells have been observed both in circulation and within tissues of T2DM patients, correlating with heightened inflammation through the secretion of various inflammatory cytokines (45). The signature cytokine secreted from the Th17 cells is IL-17A (traditionally referred to as IL-17), but they can also secrete IL-21, IL-22, IL-17 F, and granulocyte monocyte-colony stimulating factor (GM-CSF) (44). IL-17 enhances the recruitment and activation of neutrophils and macrophages, which, in turn, release additional TNF-α and IL-6, thus perpetuating chronic inflammation (44, 46) (**Figure 2A**). Research indicates that obesity selectively promotes the expansion of the Th17 T-cell lineage, which exacerbates disease in murine models of autoimmunity (47). Additionally, human studies support this concept, as clinical data suggest that IL-17 is expressed at elevated levels in obese individuals (48). Furthermore, neutralization of IL-17 and modulating Th17 cell activity has demonstrated therapeutic potential in mitigating disease symptoms across various inflammatory and autoimmune conditions, highlighting its role in mediating inflammatory responses (49, 50).

Th22 cells, which produce IL-22, have a more nuanced role in T2DM-associated inflammation. Elevated levels of Th22 cells and IL-22 have been observed in individuals with obesity and T2DM. This elevation is more pronounced than that of other pro-inflammatory subsets, such as Th1 and Th17 cells (51). IL-22 can amplify IL-1β-driven inflammation in human adipose tissue (52). In addition, IL-22 has been implicated in hepatic inflammation, which is commonly associated with insulin resistance and non-alcoholic fatty liver disease (NAFLD)—conditions that frequently co-occur with T2DM (53).

Conversely, there is a notable decrease in the numbers of anti-inflammatory T cell subsets, particularly Tregs and Th2 cells, in both peripheral blood and insulin-sensitive tissues, such as adipose tissue and skeletal muscle, in T2DM (40, 54, 55). Tregs are essential for maintaining immune homeostasis by suppressing the activity of pro-inflammatory T cells and other immune cells through the production of anti-inflammatory cytokines, including IL-10 and transforming growth factor-beta (TGF-β). In addition, Tregs express CD25, which competes for IL-2, a crucial growth factor for effector T cells. This competition limits the expansion of pro-inflammatory T cells (56, 57). Experimental models have demonstrated that the depletion of Tregs in mice results in increased tissue inflammation, while adoptive transfer of Tregs into obese mice reduces adipose tissue inflammation (58–61).

Th2 cells secrete anti-inflammatory cytokines such as IL-4, IL-5, and IL-10, which play a crucial role in modulating immune responses and mitigating inflammation. Studies have demonstrated that Th2 cells, along with serum concentrations of Th2-associated cytokines—particularly IL-4—are significantly reduced in individuals with T2DM (10, 62, 63) (**Figure 2A**). This decline impairs the immune system’s ability to counteract the pro-inflammatory environment, thereby exacerbating inflammation. Under normal conditions, Th2 cells suppress pro-inflammatory pathways to maintain immune homeostasis; however, their diminished presence in T2DM contributes to sustained inflammation, ultimately accelerating disease progression (10, 63).

There is substantial evidence that the disproportionate increase in pro-inflammatory T cells, coupled with a reduction in anti-inflammatory subsets, correlates with T2DM severity (64). Moreover, a positive association has been observed between elevated pro-inflammatory T cell counts and disease severity (64), suggesting that this imbalance in T cell subsets serves as a critical driver of T2DM pathogenesis by promoting inflammation.

### 3.2 Increased pro-inflammatory T cells directly impair insulin sensitivity

Insulin plays a key role in maintaining blood sugar by activating the phosphoinositide 3-kinase (PI3K)/Akt signaling pathway and regulating GLUT4 to enhance glucose uptake (65). Pro-inflammatory cytokines released by CD8^+^ T cells, Th1 cells, Th17 cells, and Th22 cells—including TNF-α, IFN-γ, IL-17, and IL-22—play a significant roles in impairing insulin signaling, leading to reduced glucose uptake and then increased insulin resistance. Himsworth first observed that simultaneous injections of glucose and insulin in diabetic patients still led to elevated blood sugar, a phenomenon termed insulin insensitivity (66). This reflects the core feature of insulin resistance: despite normal insulin levels, target tissues fail to adequately promote glucose uptake, utilization, and storage via GLUT4, impairing the normal hypoglycemic response and raising blood glucose (67). Compensatory increases in insulin secretion follow, ultimately resulting in systemic insulin resistance (68).

In patients with obesity and T2DM, the production of IFN-γ and TNF-α is significantly elevated (69, 70). Inhibition or deletion of IFN-γ or TNF-α in mice improves insulin sensitivity (71, 72). CD8^+^ T and Th1 cells are the primary sources of IFN-γ and TNF-α. Additionally, TNF-α is also produced by M1 macrophages in response to T cell activation. Both IFN-γ and TNF-α can inhibit insulin receptor signaling by promoting the phosphorylation and degradation of insulin receptor substrates (IRS) and downregulating GLUT4. IFN-γ mediates its effects by activating the signal transducers and activators of transcription (STAT) family via the Janus kinase (JAK) pathway through IFN-γ receptor (73, 74). Upon activation, STAT transcription factors homodimerize or heterodimerize, translocate to the nucleus, and induce the transcription of suppressor of cytokine signaling (SOCS) family members. The upregulation of SOCS1 and SOCS3 has been demonstrated in the liver, muscle, and adipose tissues of obese db/db mice and promotes the phosphorylation and degradation of IRS-1 and IRS-2, thereby impairing downstream signaling through the PI3K/Akt pathway, downregulating GLUT4, and ultimately reducing glucose uptake (75, 76). Meanwhile, TNF-α activates stress-related kinases, including p38 mitogen-activated protein kinase (MAPK), c-Jun N-terminal kinase (JNK), and inhibitor of kappa B kinase (IKK), which results in further serine phosphorylation of the insulin receptor and IRS-1, thus leading to insulin resistance on glucose uptake and GLUT4 translocation (77, 78). Furthermore, TNF-α has been shown to upregulate phosphatase and tensin homolog (PTEN), a negative regulator of the PI3K/Akt pathway. Increased PTEN activity antagonizes PI3K signaling, contributing to reduce glucose uptake (72) (**Figure 2B**).

IL-17 and IL-22, two major effector cytokines primarily produced by Th17 and Th22 cells, have also been shown to be involved in insulin resistance. The levels of circulating IL-17 and IL-22 are found to be higher in individuals with obesity and T2DM (79–81). Incubation of rat muscle strips with IL-17 or IL-22 has been shown to inhibit glucose uptake in skeletal muscle isolated from rats and reduce insulin sensitivity in cultured human hepatocytes (82). Additionally, IL-17 activates the MAPK pathway, which is associated with the development of insulin resistance. Similarly, IL-22 induces the activation of JAK1 and tyrosine kinase 2 (TYK2), promoting the translocation of STAT3 into the nucleus. Furthermore, IL-22 signaling also involves the activation of MAPK pathways, thereby contributing to reduce glucose uptake (83) (**Figure 2B**).

### 3.3 T cell subset imbalance polarizes macrophages exacerbating inflammation and insulin resistance

During the progression of T2DM, there’s notable polarization of macrophages towards the classically activated M1 type, which fosters inflammation and compromises insulin sensitivity (84, 85). This polarization is not an isolated event but is tightly regulated by immune crosstalk between macrophages and various T cell subsets. CD8^+^ T cells are particularly significant in influencing this polarization. Additionally, other T cell subsets, such as Th1, Th2, Th17 cells, Tregs, and invariant natural killer T (iNKT) cells also play critical roles in this immune-macrophage interaction.

As discussed earlier, CD8^+^ T cells infiltrate adipose tissue before the accumulation of macrophages in obese mouse models. These cells secrete chemokines such as C-C chemokine motif ligand 2 (CCL2), which are critical for recruiting monocytes from the circulation into inflamed tissues. Once recruited, these monocytes differentiate into macrophages and, under the influence of CD8^+^ T cell-derived granzyme B, IFN-γ and TNF-α, polarize towards the M1 phenotype (9). Elevated levels of CCL2 in obese mice adipose tissue have been correlated with increased macrophage infiltration and insulin resistance (86). Notably, depletion of CD8^+^ T cells in obese mice results in a significant reduction in M1 macrophage numbers within adipose tissue (9) (**Figure 2A**).

An altered Th1/Th2 balance, characterized by an increase in Th1 cells and a decrease in Th2 cells, contributes to macrophage polarization during T2DM development. M1 macrophages are polarized by Th1 cytokines such as IFN-γ, GM-CSF (37, 87, 88). In HFD mouse models, the elevated presence of Th1 cells in adipose tissue significantly correlated with increased M1 macrophages (87, 89). Moreover, mice deficient in IFN-γ or Th1 cells exhibited a reduction in M1 macrophage infiltration and improved insulin sensitivity (43, 71). This evidence indicates the pivotal role of Th1-driven macrophage polarization in T2DM pathogenesis. Conversely, M2 macrophages are anti-inflammatory and are polarized by Th2 cytokines such as IL-4 and IL-13, producing anti-inflammatory cytokines such as IL-10 and TGF-β (37) (**Figure 2A**). During the development of T2DM, however, the balance shifts toward a Th1-dominant environment, skewing macrophage polarization towards the M1 phenotype and reducing the presence of anti-inflammatory M2 macrophages (88).

Th17 cells also contribute to M1 macrophage polarization through the secretion of IL-17 (90). In HFD-fed mouse models, elevated levels of IL-17 were associated with increased numbers of M1 macrophages in adipose tissue, liver, and skeletal muscle, correlating with heightened local inflammation and systemic insulin resistance (44, 91). Blocking IL-17 with neutralizing antibodies resulted in a significant reduction of M1 macrophage markers, decreased pro-inflammatory cytokine production, and improved insulin sensitivity in mouse disease models (92). Moreover, *in vitro* studies have shown that macrophages exposed to IL-17 exhibit enhanced M1 polarization (92). Furthermore, IL-17 has been shown to influence macrophage autophagy through the PINK1/Parkin pathway, further promoting M1 polarization. Inhibition of IL-17 activity in autoimmune disease rats experimental models resulted in reduced autophagy and a shift away from the M1 phenotype (93) (**Figure 2A**). These IL-17-stimulated macrophages displayed a heightened inflammatory response when co-cultured with adipocytes, leading to increased adipocyte insulin resistance and lipid accumulation (94).

Additionally, Tregs and M1 macrophages exhibit a trade-off relationship, where a decreased frequency of Tregs leads to the accumulation of M1 macrophages (95, 96). Consistently, a reduction in Tregs leads to the accumulation and polarization of M1 macrophages in the development of T2DM (95). Strategies targeting Tregs to restore their numbers, as well as the adoptive transfer of Tregs, have been shown to decrease macrophage infiltration and significantly reduce the classical activation of M1 macrophages (95, 97, 98). In addition, recent research has unveiled that Tregs can steer macrophages towards the M2 polarization by releasing IL-10 and soluble fibrinogen-like protein 2 (sFGL2) (99). iNKT cells have similarly been implicated in regulating macrophage polarization in obesity-related metabolic dysfunction. In both obese mouse models and human subjects, adipose tissue iNKT cell numbers are significantly reduced, with levels inversely correlated with insulin resistance and blood glucose concentrations (100, 101). Activation of adipose-resident iNKT cells can improve systemic glucose homeostasis and enhance M2 macrophage polarization in adipose tissue (101).

Overall, the imbalance of T cell subsets is a key driver of macrophage recruitment and polarization toward the pro-inflammatory M1 phenotype, which not only amplifies tissue-specific inflammation but also sustains systemic low-grade chronic inflammation and insulin resistance, thereby accelerating the progression to T2DM.

### 3.4 T cell subset imbalance promotes islet inflammation and β-cell dysfunction

Islet inflammation and subsequent islet β-cell dysfunction are key pathogenic elements of T2DM (102). Studies have demonstrated a notable increase in T cell infiltration and T cell imbalance within the islets of T2DM patients and corresponding animal models (103–105).

TNF-α and IFN-γ, secreted by CD8^+^ T cells, directly contribute to β-cell dysfunction and apoptosis (106). Elevated TNF-α levels in T2DM were associated with increased β-cell apoptosis and reduced insulin secretion, linking this cytokine to the deterioration of β-cell function (107). TNF-α was reported to induce β-cell apoptosis through the activation of death receptors and the nuclear factor κB (NF-κB) signaling pathway (102, 106). Earlier research demonstrated that TNF-α inhibits insulin secretion by reducing glucose-stimulated calcium influx in β-cells (108). This inhibition is possibly mediated through the activation of stress and inflammatory signaling pathways, including JNK, p38 MAPK, and NF-κB (108). IFN-γ induces apoptosis in β-cells by activating the JAK-STAT signaling pathway and enhances the expression of MHC class I molecules on β-cells, making them more susceptible to attack by cytotoxic CD8^+^ T cells (106, 109). As a result, IFN-γ creates a feedback loop that perpetuates inflammation and accelerates β-cell destruction. Furthermore, macrophages within the islets undergo significant phenotypic changes during the progression of T2DM, shifting from an anti-inflammatory M2 phenotype to a pro-inflammatory M1 phenotype (110, 111). This transition can be influenced by the local inflammatory milieu, including cytokines secreted by infiltrating T cells, particularly CD8^+^ T cells. CD8+ T cells are markedly increased within the islets of T2DM patients, where they secrete granzyme B and IFN-γ, both of which promote M1 polarization and contribute to β-cell cytotoxicity (112). The interaction between CD8^+^ T cells and macrophages in the islets of T2DM requires further investigation; however, the co-occurrence of CD8^+^ T-cell infiltration and an increase in M1 macrophages has been consistently observed in the islets of T2DM (35). The increased CD8^+^ T-cell infiltration within the pancreatic islets creates a toxic microenvironment for β-cells via the secretion of TNF-α and IFN-γ and the induction of M1 macrophage polarization, ultimately leading to β-cell dysfunction and apoptosis. This detrimental cycle exacerbates insulin secretion deficiency and worsens hyperglycemia in T2DM (**Figure 2C**).

A shift in the Th17/Tregs balance occurs during the progression of T2DM, characterized by a relative decrease in Tregs and an increase in Th17 cells within pancreatic islets (105). While the precise mechanisms linking Th17/Treg imbalance to β-cell dysfunction remain under investigation, the existing evidence strongly suggests that the increase M1 macrophages and pro-inflammatory cytokines which induced by Th17/Treg imbalance, contribute to β-cell damage (44, 90, 95, 105) (**Figure 2C**). Importantly, restoring the Th17/Treg balance holds promise in preserving β-cell function and improving metabolic outcomes in T2DM (105). In addition to Th17/Treg imbalance, the Th1/Th2 balance also plays a critical role in the regulation of pancreatic islet inflammation. Studies in diabetes-prone (DP) rats have shown that the mRNA expression of the Th1 cytokine IFN-γ is significantly elevated within pancreatic islets, and this increase is positively correlated with both inflammatory infiltration and β-cell destruction (113). In contrast, the Th2 cytokines IL-4 and IL-10 can effectively reduce β-cell death and injury induced by pro-inflammatory and Th1 cytokines, indicating an anti-inflammatory and protective role of Th2 responses (114). Furthermore, other studies observed increased Th22 cell numbers in patients with insulin-resistant T2DM and an inverse correlation with clinical functional measures of pancreatic β-cells (51). This finding preliminarily suggests a role for Th22 cells in β-cell dysfunction, although the potential mechanisms have not yet been elucidated.

In all, T cell subsets within the pancreatic islets undergo significant changes during T2DM, characterized by an increase in pro-inflammatory T cells and a decrease in Tregs and Th2 cells. These shifts, along with alterations in macrophage polarization, create a pro-inflammatory microenvironment that promotes islet inflammation and β-cell dysfunction.

## 4 T2DM complications associated with T cell subset imbalance

In the advanced stages of T2DM, chronic hyperglycemia, insulin resistance, and systemic inflammation drive the development of severe complications such as AS, DKD, and DR. Increasing evidence indicates that T cell subset imbalance and tissue infiltration—particularly by Th1, Th17, CD8^+^ T cells, and Tregs, play critical roles in the progression of these complications. Understanding the roles of T cell subsets in these diabetic complications is crucial for developing targeted therapies aimed at modulating immune responses to prevent or mitigate disease progression (**Table 2**).

**Table 2:** T2DM complications associated with T cell subset imbalance.

T cell subset	Cytokine	Affected tissue	Pathological outcome
CD8^+^ T cells	TNF-α, IFN-γ, granzyme A/B, Prf	retina	neovascularization, vascular dysfunction, retinal inflammation and damage→DR
Th1 cells	IFN-γ	vascular, kidney, foot	vascular cell apoptosis, extracellular matrix degradation, vascular inflammation, hypertriglyceridemia, inhibit VSMC proliferation, plaque vulnerability→AS; kidney inflammation and damage→DKD; extracellular matrix degradation, poor healing→DFU
Th17 cells	IL-17	vascular, kidney, retina, foot, central nervous system	vascular inflammation, plaque vulnerability→AS; kidney inflammation and function→DKD; RMC dysfunction, neovascularization, retinal inflammation and damage→DR; wound inflammation and poor healing→DFU; neuronal death, neurinflammation, cognitive decline→AD
Tregs	IL-10, TGF-β	kidney, retina, foot	inhibit kidney inflammation⟞DKD; inhibit Retinal inflammation⟞DR; inhibit wound inflammation⟞DFU

### 4.1 T cell subset imbalance is involved in the process of AS in T2DM

In T2DM, there is an increased infiltration of pro-inflammatory T cell subsets, particularly Th1 and Th17 cells, into atherosclerotic plaques, this infiltration contributes to the progression of AS, a common complication in T2DM patients (16). The chronic inflammatory environment in T2DM leads to the increased differentiation of CD4^+^ T cells into Th1 and Th17 cells, which amplifying the inflammatory cascade within the plaques (16, 17).

**Figure 3:**
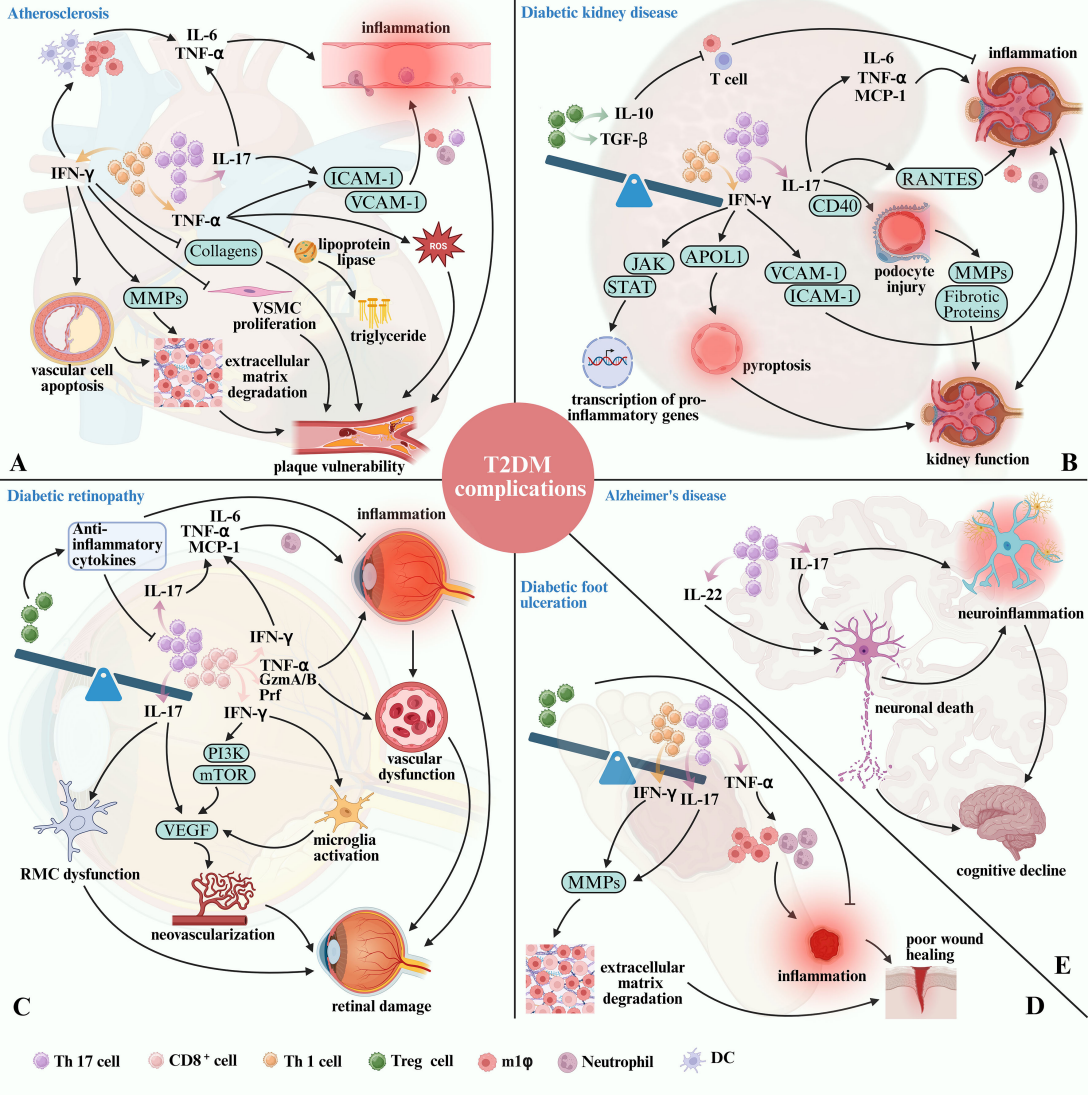
The role of T cell subset imbalance in T2DM-related complications. **(A)** IFN-γ secreted by Th1 cells promotes the development of AS and the instability of atherosclerotic plaque by causing inflammation, degradation of extracellular matrix, and inhibiting the proliferation of VSMC and collagens expression. At the same time, TNF-α secreted by Th1 cells causes inflammation, increases ROS and accumulates triglyceride, eventually aggravate AS. Th17 cells contribute to plaque vulnerability by enhancing pro-inflammatory cytokine production, and upregulating adhesion molecules via IL-17. **(B)** Elevated Th1 and Th17 activity contributes to persistent renal inflammation, immune cell infiltration, and direct cellular injury. In contrast, Tregs deficiency impairs immune regulation, thereby exacerbating chronic kidney damage and fibrosis. **(C)** Th17 cells promote retinal inflammation and pathological neovascularization through IL-17 production, while CD8^+^ T cells aggravate vascular permeability and retinal injury by secreting IFN-γ and TNF-α. Conversely, the reduction of Tregs aggravates inflammation and inhibits tissue repair. **(D)** Th1 and Th17 cells increase MMPs activity and stimulate the infiltration of neutrophils and pro-inflammatory M1 macrophages via TNF-α secretion, thereby contributing to delayed wound healing and the progression of DFU. While the decrease of Tregs boosts the occurrence of inflammation. **(E)** Th17 cells may exacerbate neuroinflammation and neuronal damage in T2DM through the secretion of IL-17 and IL-22, potentially increasing the risk of AD in diabetic individuals.

Th1 cells are key mediators in plaque inflammation due to their secretion of IFN-γ and TNF-α (115). IFN-γ plays a significant role in the development and progression of atherosclerotic plaques through several mechanisms. Firstly, IFN-γ promotes the recruitment of additional immune cells, such as macrophages and DCs, to the site of the plaque, promoting the release of additional TNF-α and IL-6, thereby amplifying the local inflammatory response (17, 115). Furthermore, IFN-γ accelerates apoptosis of vascular cells within plaques and induces the release of matrix metalloproteinases (MMPs). This process leads to extracellular matrix degradation, weakening the structural integrity of plaques and making them more prone to rupture or erosion (115, 116). Additionally, IFN-γ inhibits vascular smooth muscle cell proliferation and suppresses the expression of collagen genes, such as collagen I and III, thereby impairing the repair and stability of the fibrous cap covering the plaque, increasing the risk of plaque rupture (115, 116) (**Figure 3A**). The above detrimental effects of IFN-γ on atherosclerotic plaque development and stability are further supported by murine studies. Daily injections of recombinant IFN-γ in ApoE^-/-^ mice resulted in a twofold increase in atherosclerotic lesion size, despite a 15% reduction in plasma cholesterol levels (117). Conversely, ApoE^-/-^ mice lacking IFN-γ or its receptor exhibited significantly smaller atherosclerotic lesions compared to ApoE^-/-^ controls (118). Furthermore, IFN-γ-deficient mice demonstrated increased collagen content within the fibrous cap of plaques, indicating enhanced plaque stability (117, 118).

In addition to IFN-γ, TNF-α contributes to the pathogenesis of AS by promoting the infiltration of circulating inflammatory cells into atherosclerotic plaques. This process occurs through the upregulation of adhesion molecules, such as vascular cell adhesion molecule-1 (VCAM-1) and intercellular adhesion molecule-1 (ICAM-1), on the surface of endothelial cells, thereby perpetuating the inflammatory cycle and contributing to plaque instability (119, 120). Additionally, TNF-α suppresses lipoprotein lipase activity and reduces the oxidative metabolism of fatty acids, resulting in hypertriglyceridemia. This metabolic disturbance, coupled with TNF-α-induced production of ROS, further promotes the development of AS (121).

Th17 cells, through the secretion of IL-17, act on endothelial cells, smooth muscle cells, and macrophages within the vascular wall. Elevated IL-17 levels have been observed in atherosclerotic plaques of diabetic patients and animal models, correlating with increased inflammatory cell infiltration and plaque instability (44, 122, 123). IL-17 enhances the production of TNF-α and IL-6, by endothelial and smooth muscle cells within the vascular wall (16, 124). Besides, IL-17 induces VCAM-1 expression, facilitating immune cells adhesion and transmigration into the arterial wall (125). This cytokine-induced milieu promotes the recruitment of macrophages, neutrophils and additional Th17 cells to the plaque, further exacerbating inflammation (123). Macrophages and neutrophils, in turn, release ROS and proteases that further damage the vascular wall, contributing to plaque vulnerability (126). The hyperglycemic and altered hormonal environment characteristic of T2DM fosters Th17 differentiation, with the presence of Th17 cells in atherosclerotic lesions being strongly associated with reduced collagen content and heightened metalloproteinase activity (44). This activity degrades the extracellular matrix, weakening the fibrous cap of plaques, thereby increasing the risk of rupture (44). Research indicates that Th17 cells, are present in higher percentages in hyperlipidemic patients compared to healthy donors. In mouse models, neutralization of IL-17 resulted in a reduction of VCAM-1 expression, decreased infiltration of macrophages and neutrophils, and smaller atherosclerotic lesions, highlighting the pro-atherogenic role of Th17 cells in promoting inflammation under hyperlipidemic conditions (123) (**Figure 3A**).

In summary, increased infiltration of pro-inflammatory Th1 and Th17 cells into atherosclerotic plaques significantly contributes to plaque progression and instability.

### 4.2 T cell subset imbalance regulates the progression of DKD in T2DM

DKD, a severe and progressive complication of T2DM, is characterized by structural and functional changes in the kidneys, ultimately leading to chronic kidney disease and end-stage renal disease. One of the hallmark features of DKD is the infiltration of immune cells, particularly T cells, into the renal tissue (18, 19). CD4^+^ T cells, particularly Th1 and Th17 cells, play a crucial role in the pathogenesis of DKD, with their increased presence correlating with the severity of renal damage (18, 19, 127). Histological analyses of kidney tissues from patients with DKD reveal that elevated Th1 and Th17 cell levels are strongly associated with significant glomerular and tubulointerstitial damage (21, 128). This damage manifests as increased mesangial expansion, thickening of the glomerular basement membrane, and interstitial fibrosis, which are key pathological features of DKD (21, 128). As the levels of Th1 and Th17 cells increase, there is a corresponding rise in IFN-γ and IL-17.

Increased circulating IFN-γ levels have been linked to alterations in kidney transcriptomic programs and progression to end-stage kidney disease in human DKD (129). IFN-γ activates the JAK-STAT pathway, leading to the transcription of pro-inflammatory genes (130). In addition to amplifying local inflammation, IFN-γ induces the expression of adhesion molecules, such as ICAM-1 and VCAM-1, on endothelial cells, thereby facilitating the recruitment of inflammatory cells, particularly macrophages, into the kidney (131). Furthermore, IFN-γ primes renal cells for pyroptosis a form of programmed cell death associated with inflammation—by inducing the expression of genes like Apolipoprotein L1 (APOL1) (**Figure 3B**). This mechanism contributes to endothelial injury and accelerates renal disease progression (130).

Patients with DKD exhibit higher levels of IL-17 compared to healthy controls (132). IL-17 promotes the release of additional pro-inflammatory mediators, including Monocyte Chemoattractant Protein-1 (MCP-1), IL-6, and TNF-α. These cytokines exacerbate inflammation within the renal interstitium and glomeruli, contributing to a vicious cycle of immune activation and tissue injury (128, 133). In mouse model, IL-17 administration significantly upregulated the expression of MCP-1 and CCL5, enhancing the recruitment of inflammatory cells to the renal tissue (134). Conversely, administration of a neutralizing anti-IL-17 antibody in leptin-deficient BTBR ob/ob mice resulted in the inhibition of NF-κB activation (135). Similarly, IL-17 neutralization reduced pro-inflammatory gene expression and inflammatory cell infiltration in an angiotensin II-induced mouse renal damage model (136). Podocyte injury is critical in DKD progression due to their role in maintaining the glomerular filtration barrier. Recent evidence suggests that Th17 cells contribute to podocyte dysfunction through both direct and indirect mechanisms (133). In a study by Zhai et al., IL-17 was shown to directly affect podocyte cytoskeletal structure in kidney disease patients, promoting foot process effacement, a precursor to proteinuria (137). *In vitro* studies have demonstrated that IL-17 increases the expression of IL-6 and TNF-α in podocytes, particularly under high-glucose conditions (138). Moreover, IL-17 binding to CD40a co-stimulatory protein upregulated on podocytes in DKD-drives glomerulosclerosis and podocyte injury by synergizing with CD40 to enhance MMPs and fibrotic protein expression (139). Furthermore, the administration of IL-17 neutralizing antibodies has been shown to reduce glomerular injury, preserve podocyte numbers, and ameliorate proteinuria in experimental models of diabetic nephropathy (127, 128, 140). Therefore, the potential of IL-17 as a treatment is significant but requires further clinical validation.

While Th1 and Th17 cells drive pro-inflammatory responses in DKD, Tregs act as a counterbalancing force, mitigating excessive inflammation and tissue damage (141). Tregs exert their protective effects through the secretion of IL-10 and TGF-β, which suppress the activity of effector T cells and macrophages (141) (**Figure 3B**). However, in the context of T2DM and DKD, the number and function of Tregs are often compromised, tipping the balance towards a pro-inflammatory state (142, 143). Eller et al. (144) provided compelling evidence of the protective role of Tregs in DKD. In their study, depletion of Tregs in diabetic mice led to exacerbated kidney damage, demonstrated by increased urine albumin-to-creatinine ratios, enlarged glomerular diameters, and intensified mesangial matrix expansion (144). Conversely, the adoptive transfer of Tregs into diabetic mice was shown to attenuate these pathological changes, highlighting the potential therapeutic benefits of Tregs modulation in DKD (144).

The dynamic interplay between Th1, Th17, and Tregs significantly influences the progression of DKD. Increased Th1 and Th17 responses contribute to sustained renal inflammation, immune cell activation, and direct cellular injury, while a deficiency in Tregs fails to provide adequate immune regulation. Collectively, these alterations in T cell subsets drive the initiation and progression of kidney damage in DKD.

### 4.3 T cell subset imbalance contributes to the development of DR in T2DM

DR is the leading cause of blindness in adults aged 24–70 years and ultimately occurs in approximately one-third of individuals with T2DM (145). Recent studies have implicated T cells in the development of DR, noting T-cell infiltration in the retina of affected patients.

Th17 cells are known to drive key pathological processes in DR, including inflammation and angiogenesis, which contribute significantly to retinal damage and vision loss (146). Th17 cells exert their effects primarily through the secretion of IL-17 that exacerbates retinal inflammation and promotes pathological angiogenesis (147). IL-17 amplifies the inflammatory environment within the retina by stimulating the release of additional IL-6, TNF-α, and chemokines like MCP-1, creating a cascade that further recruits inflammatory cells to the retinal microenvironment (148–151). This cytokine-driven inflammation disrupts the blood-retinal barrier, leading to increased vascular permeability and microvascular damage, hallmark features of DR (152–154). Experimental evidence on mice indicates that IL-17 exacerbates hyperglycemia-induced activation and dysfunction of retinal Müller cells (RMCs), which provide essential structural, metabolic, and homeostatic support to retinal neurons while also serving as key modulators of retinal immune and inflammatory responses (147, 155, 156). Dysfunction of RMCs disrupts their ability to support neuronal and vascular health, thereby contributing to the pathological processes observed in DR (147, 156). Retinal microvascular damage and RMCs abnormalities are reduced in IL-17 knockout diabetic mice (147, 156). Furthermore, IL-17-deficient mice display a significant reduction in inflammatory cell infiltration within the retina and lower levels of angiogenesis-related cytokines, such as Vascular endothelial growth factor (VEGF), which plays a central role in neovascularization and the progression of DR (147, 157) (**Figure 3C**). The ability of Th17 cells to promote abnormal blood vessel growth, combined with their pro-inflammatory effects, positions them as critical drivers of DR progression, particularly in the advanced stages of the disease where neovascularization poses a significant risk for vision loss.

Recent studies have also highlighted the significant role of CD8^+^ T cells in DR, particularly in promoting pathological angiogenesis and retinal damage (20, 158). CD8+ T cells have been shown to contribute to ocular neovascularization and inflammation, thereby exacerbating retinal conditions such as DR (20). Deliyanti and colleagues reported that CD8^+^ T cells directly promote pathological angiogenesis in ocular neovascular diseases, including oxygen-induced retinopathy, through the secretion of TNF-α and IFN-γ, as well as cytotoxic molecules like perforin (Prf) and granzyme A/B (GzmA/B) (20). IFN-γ plays a critical role in ocular angiogenesis, particularly by enhancing VEGF levels. In human retinal pigment epithelial cells, IFN-γ increases VEGF expression through the PI3K/mTOR pathway, which is known to promote angiogenesis and increase choroidal neovascularization (159). Additionally, IFN-γ can influence the proliferation and activation of retinal microglia—the resident immune sentinels of the retina responsible for tissue surveillance and repair, which further exacerbate local inflammation and neovascularization through the release of factors such as VEGF, TNF-α, IL-1, and IL-6 (160–163) (**Figure 3C**). TNF-α is another key mediator by which CD8^+^ T cells drive retinal vascular injury. Elevated TNF-α levels have been observed in the serum of children and adults with DR, indicating a strong correlation between TNF-α and disease severity (164, 165). Moreover, adoptive transfer experiments with CD8^+^ T cells deficient in TNF-α, IFN-γ, Prf, or GzmA/B into immunocompetent Rag1^-/-^ mice revealed that these molecules are critical mediators of retinal vascular pathology. Notably, TNF-α was found to influence all aspects of vascular pathology (20). Earlier study demonstrated that TNF-α significantly contributes to the adhesion of leukocytes to retinal vasculature, a process known as leukostasis. This adhesion can lead to capillary occlusion and subsequent ischemic damage (166). TNF-α-deficient mice exhibit reduced leukostasis in models of ischemic retinopathy. In addition, TNF-α increases vascular permeability by disrupting the blood-retinal barrier, resulting in edema. Inhibition of TNF-α has been observed to decrease such leakage, highlighting its role in maintaining vascular integrity (166).

In contrast to Th17 and CD8^+^ cells, Tregs play a protective role in DR by limiting inflammation and promoting tissue repair, which are crucial in preventing disease progression and preserving retinal function (167). Reduced numbers or dysfunction of Tregs can exacerbate DR progression, as evidenced by studies showing that Tregs depletion led to increased retinal vascular leakage, enhanced inflammatory cell infiltration, and greater neuronal loss compared to control groups in a murine model of DR (167). Conversely, the adoptive transfer of Tregs significantly improved retinal morphology and function, as evidenced by reduced vascular leakage, enhanced preservation of the photoreceptor layer, and decreased signs of gliosis (167, 168). These findings underscore the critical role of Tregs in maintaining the structural and functional integrity of the retina in DR. Tregs may achieve this protective effect through the secretion of anti-inflammatory cytokines, which suppress the activation of pro-inflammatory immune cells, including Th17 and CD8^+^ cells (169). This suppression helps to control the chronic inflammatory environment that contributes to retinal damage in DR (167, 168). Beyond their anti-inflammatory functions, Tregs also support tissue repair processes through the activation of Müller cells (167, 170) (**Figure 3C**), the principal glial cells responsible for maintaining retinal structure and function, by reducing oxidative stress and promoting the release of neurotrophic factors that support neuronal survival (170, 171). Tregs further aid in the repair of the retinal vasculature by regulating angiogenesis, stabilizing endothelial cells, and promoting controlled vascular remodeling (167, 168). These combined actions help maintain the integrity of the retinal tissue, reduce vascular leakage, and prevent further damage, highlighting Tregs as a potential therapeutic target in DR management.

Taken together, these studies suggest that an imbalance in T cell subsets significantly contributes to the pathogenesis of DR by promoting retinal inflammation and angiogenesis. Th17 cells contribute to retinal inflammation and neovascularization through the production of IL-17, while CD8^+^ T cells exacerbate retinal damage and vascular permeability through the secretion of TNF-α and IFN-γ. Tregs, on the other hand, play a protective role by limiting inflammation and promoting tissue repair (**Figure 3C**).

### 4.4 T cell subset imbalance drives the pathogenesis of diabetic foot ulceration

Diabetic foot ulceration (DFU) is a severe and debilitating complication of T2DM, characterized by chronic, non-healing wounds. The impaired healing observed in DFU is strongly linked to T cell dysfunction and an imbalanced immune response. In the context of DFU, there is a notable imbalance between pro-inflammatory T cell subsets, such as Th1 and Th17, and Tregs, which significantly impairs the wound healing process (140, 172, 173). At the site of DFU, there is an increased presence of pro-inflammatory T cells, particularly Th1 and Th17 cells. These cells secrete IFN-γ, IL-17, and TNF-α (172, 173). The elevated levels of these cytokines promote the recruitment and activation of neutrophils and M1 macrophages, leading to a persistent inflammatory state (172). This prolonged inflammation creates a hostile wound environment that delays the resolution of inflammation and the initiation of tissue repair processes (174). Additionally, the combined actions of IFN-γ and IL-17 boost MMP activity, which degrades the extracellular matrix, further hindering wound closure (172). Therapeutic strategies neutralizing IL-17 and IFN-γ activity have shown promise in reducing MMP expression and improving healing outcomes in chronic wounds (175, 176). Conversely, there is a marked decrease in Tregs in DFU patients. This reduction in Tregs results in a loss of immune regulation, allowing for an uncontrolled inflammatory response dominated by Th1 and Th17 cells (**Figure 3D**).

The lack of Treg-mediated suppression contributes to the chronic inflammation that is characteristic of DFU, resulting in poor wound healing outcomes (140, 177). An *in vivo* study demonstrated that enhancing Tregs function improved wound healing and reduced inflammation in diabetic mice, highlighting the critical role of Tregs in regulating the immune response during wound repair (140, 177).

### 4.5 Alzheimer’s disease is associated with T cell subset imbalance

Clinical studies have shown that patients with T2DM or impaired glucose metabolism have a significantly higher risk of developing Alzheimer’s disease (AD) compared to normoglycemic individuals (178, 179). Similarly, elevated levels of inflammatory mediators have been consistently observed in the brains of AD patients, suggesting that chronic inflammation may serve as a shared pathological mechanism linking T2DM and AD (180, 181).

One key feature common to both conditions is the imbalance of T cell subsets, particularly involving pro-inflammatory Th17 cells. Patients with AD exhibit increased numbers of IL-17-secreting CD4^+^ T cells, reflecting a similar T cell subset imbalance observed in T2DM (182–184). This suggests that Th17 cells and their downstream cytokines may play a central role in AD pathogenesis. In experimental models of AD, such as intrahippocampal Aβ1-42-injected rats, Th17 cells have been shown to traffic from the circulation to the central nervous system (CNS) (185). This process is associated with a disrupted blood-brain barrier, which allows Th17 cells and their cytokines, IL-17 and IL-22, to infiltrate the hippocampus (185). The presence of these pro-inflammatory cytokines in the CNS contributes to neuronal death and cognitive decline (186, 187). Th17 cells also promote neuroinflammation through the release of IL-17, which exacerbates the inflammatory milieu in the brain (186, 187) (**Figure 3E**). This inflammation can lead to further neuronal damage and is implicated in the progression of cognitive impairments characteristic of AD (186, 187). Mouse models of AD and T2DM have demonstrated that using IL-17 inhibitors can reduce neuroinflammation, Aβ deposition, and cognitive decline (188).

## 5 Immunotherapeutic strategies targeting T cell subset imbalance

Although lifestyle intervention combined with oral hypoglycemic drugs (such as sulfonylureas and thiazolidinediones) remains the cornerstone of diabetes treatment, tight blood glucose control alone is insufficient to reduce all-cause mortality or prevent complications in patients with type 2 diabetes and carries a risk of hypoglycemia (189, 190). Long-term medication can also cause anxiety, treatment fatigue, and other issues, significantly reducing quality of life (191). Therefore, current therapeutic strategies for T2DM are expanding from traditional glycemic control toward modulation of immune and metabolic pathways.

This review emphasizes the central role of T cell subset imbalance in the progression and complications of T2DM, offering new perspectives for prevention and treatment. From the above discussion, it is evident that the imbalance between anti-inflammatory and pro-inflammatory T cell subsets is a key driver of diabetes progression and the development of its complications. Numerous studies have demonstrated that restoring the balance of T cell subsets can alleviate both diabetes and its related complications. For example, berberine (BBR) has been shown to regulate the key transcription factors RORγt and Foxp3 in T cells, restore the balance between Th17 and Tregs, suppress inflammation, and prevent retinal damage (192). In clinical settings, BBR is frequently used as an adjunct to conventional glucose-lowering agents and has demonstrated efficacy in improving glycemic control, lipid metabolism, and insulin sensitivity in patients with T2DM (193). However, the bioavailability of BBR remains notably low. Moreover, both preclinical and clinical studies have employed heterogeneous dosages, delivery methods, and treatment durations, leading to substantial variability in observed outcomes (194–196). Consequently, the lack of a standardized formulation and dosing protocol represents a significant barrier to its broader clinical translation and regulatory approval. Additionally, in the early stages of disease in db/db mice, co-injection of recombinant mouse IL-2 and anti-IL-2 monoclonal antibody can expand Treg subsets, reduce activation of inflammasomes, prevent retinal neurodegeneration, and mitigate DR (197). Moreover, low-dose IL-2 administration has been shown to expand Tregs and reduce the release of pro-inflammatory cytokines, thereby controlling inflammation and alleviating DR and severe vascular injury (198). Similarly, low-dose IL-2 therapy has been reported to expand Tregs and restore cognitive function in mice (199), offering a potential therapeutic approach for diabetes-related AD. In clinical settings, low-dose IL-2 has shown promise in expanding Tregs and correcting immune imbalances in patients with T1DM (200). However, IL-2 dosing must be strictly controlled, as it is intrinsically a pro-inflammatory cytokine, and excessive administration may exacerbate inflammation (201). Furthermore, overactivation or expansion of Tregs could lead to broad immunosuppression, potentially increasing susceptibility to infections and cancer (202, 203). Treatment with anti-CD8 antibodies can block CD8^+^ T cell infiltration into injured tissues and decrease the release of pro-inflammatory factors after ischemic injury in T2DM mice, thereby promoting vascular regeneration in damaged tissues (204), which may provide new directions for the treatment of diabetic complications such as AS and DR. Currently, anti-CD8 monoclonal antibody therapy is still in the early stages of clinical trials. It has been shown to reduce relapse rates and improve overall survival in patients with acute leukemia following bone marrow transplantation; however, its approved use is currently restricted to acute leukemia (205). Moreover, the high production costs and complex manufacturing requirements of this monoclonal antibody restrict its broad clinical prospects (206). Collectively, these findings indicate that restoring T cell subset balance plays a critical role in treating diabetes and its complications; however, the clinical application of these approaches remains limited and requires further investigation.

This review also highlights that an increase in pro-inflammatory cytokines and a decrease in anti-inflammatory cytokines, driven by an imbalance in T cell subsets, play critical roles in regulating the onset and progression of T2DM and its complications, suggesting that modulation of cytokine profiles could offer therapeutic potential for controlling disease progression. For instance, the traditional Chinese medicine Huayuwendan Decoction (HYWD) can reduce serum levels of IL-6, TNF-α, IL-17, and IL-1β, inhibit the IL-17/NF-κB signaling pathway, and improve DKD in rats (207). Clinical evidence indicates that this traditional Chinese medicine component can significantly lower fasting blood glucose levels and ameliorate insulin resistance in metabolic syndrome patients. With its gentle action profile and low incidence of adverse effects, it shows significant promise for broad clinical implementation (208). And it has long been used to treat diabetes in clinical practice (209). Additionally, in diabetic mouse models, treatment with therapeutic antibodies, such as subcutaneous injection of adalimumab, which inhibits TNF-α and received approval in 2008 for the treatment of multiple pediatric conditions (such as rheumatology, gastroenterology and dermatology), can accelerate wound healing (210, 211). Nevertheless, when used for different indications, serious adverse events including infections, nasopharyngitis, and headaches have been reported (212). Overexpression of the anti-inflammatory cytokine IL-10 can protect adipocytes in insulin-sensitive tissues from TNF-α–induced insulin resistance, thereby controlling diabetes progression (213). Early clinical trials investigated the use of recombinant IL-10 protein for the treatment of rheumatoid arthritis (RA), psoriasis, and Crohn’s disease (CD), but with limited effectiveness (214). Novel IL-10 *in vivo* expression strategies have shown promising efficacy against relapsed/refractory B-cell acute lymphoblastic leukemia (B-ALL) in early trials, though broader clinical applications require further exploration (215, 216). But there is a potential risk of immunosuppression as an adverse effect (215). Sitagliptin, widely used in clinical practice, treats T2DM by reducing β-cell apoptosis induced by the pro-inflammatory cytokine IL-1β (217). Sitagliptin remains an important option in the management of T2DM patients due to its convenient oral administration, low potential for pharmacokinetic drug interactions, and favorable efficacy, tolerability, and safety profile (218–221). However, there are still side effects of short-term headaches (222). Moreover, increased IFN-γ expression has been observed in Hif-1α-deficient CD4^+^ T cells cultured under high-glucose conditions, accelerating DKD progression, suggesting that targeting the high glucose/Hif-1α transcription axis may help prevent DKD by limiting IFN-γ secretion from Th1 cells (223). Interestingly, a genetic association study in diabetic patients and mice demonstrated that the HIF-1α Pro582Ser polymorphism exhibits a negative correlation with the risk of diabetic nephropathy, suggesting a protective function (224). Therefore, future pharmacological strategies employing agonists that specifically target HIF-1α activation may represent a promising approach for developing preventive drugs against DKD.

In addition, macrophage polarization driven by T cell subset imbalance plays a crucial role in the progression of diabetes and its complications, and research into regulating the shift from pro-inflammatory M1 macrophages to anti-inflammatory M2 macrophages as a therapeutic strategy for diabetes is increasing. Studies have shown that an IL-17-blocking antibody can inhibit M1 macrophage activity and accelerate diabetic mice wound healing (225). IL-17 blocking antibodies have been successfully used in the clinical treatment of autoimmune diseases such as psoriasis, psoriatic arthritis, and ankylosing spondylitis, but are still in early clinical trials (226). However, long-term use may cause adverse reactions such as mental disorders, infections, nasopharyngitis, and injection site reactions (227). Similarly, local subcutaneous injection of recombinant IL-25 (IL-17E) into diabetic mice wounds can promote macrophage polarization toward the M2 phenotype through the PI3K/Akt/mTOR signaling pathway, thereby releasing pro-healing cytokines and promoting wound repair (228). IL-25 treatment has been shown to work in mouse models, but whether it can have the same effect in the clinic requires further testing (229). Moreover, IL-25 is involved in various immune modulations, and improper use may lead to immune disorders (230). Recent studies have demonstrated that lipid nanoparticles containing trisulfide bonds can induce IL-4 expression by Tregs, thereby promoting M2 macrophage polarization at wound sites and significantly accelerating diabetic wound healing (231). Numerous lipid nanoparticle-based formulations are undergoing clinical trials for the treatment of infectious diseases, cancers, and genetic disorders. However, concerns persist regarding their potential for accumulation within the body (232). Newly developed cLpT@siRNA nanocomposites, based on antioxidants and cationic lipids, can effectively eliminate excessive ROS, promote the polarization of M1 to M2 macrophages, and inhibit MMP9 gene expression in macrophages, thereby significantly accelerating diabetic wound healing and promoting angiogenesis and collagen deposition (233). As a new type of biomaterial, cLpT@siRNA nanocomposites have been shown to have good compatibility *in vivo* and no *in vivo* organ toxicity in mice (231, 233). However, its clinical application still needs to be explored. These strategies offer promising therapeutic approaches for treating AS and diabetic foot ulcers and hold significant potential for clinical translation. Furthermore, some studies have shown that injecting mesenchymal stem cells (MSCs) into mice can induce M1-to-M2 macrophage polarization in skin wounds, thereby accelerating wound repair, which also represents a novel approach for diabetic wound treatment (234). Numerous clinical trials confirm MSCs treat diverse diseases, including T1DM, pulmonary dysfunction, neurological disorders, endocrine/metabolic diseases, skin burns and cardiovascular conditions (235, 236). However, in T2DM patients, metabolic dysfunction impairs autologous MSCs efficacy, favoring allogeneic sources (237). Although MSCs show broad therapeutic potential, their clinical application faces challenges including donor heterogeneity, safety risks, as well as high costs and lack of standardized protocols (236).

All the above studies demonstrate that a promising therapeutic strategy for T2DM and its complications involves correcting T cell subset imbalance, regulating inflammatory cytokines, and altering macrophage polarization. Nevertheless, the translation of some strategies to the clinic is hindered by limited applicability and potential safety concerns (**Table 3**). Importantly, lifestyle modification continues to be the foundation of diabetes management (238). In the future, combining healthy lifestyle interventions with conventional treatments and innovative immunotherapies may pave the way for more effective diabetes management.

**Table 3:** Immunotherapeutic strategies.

Immunotherapy	Specific methods	Clinical applicability	the current status of clinical trials	Potential safety concerns
Restore T cell subset balance	BBR restores Treg/Th17 balance (192)	T2DM	BBR combines with glucose-lowering drugs(such as pioglitazone and metformin) has entered clinical trials in patients with T2DM (193).	Low bioavailability, unknown optimal therapeutic dose, theoretical risk of immune overactivation (194–196)
	Low-dose IL-2 administration expands Tregs (197–199)	T1DM; Potential to treat T2DM	T1DM: Low-dose IL-2 therapy is undergoing clinical trials in T1DM patients (200). T2DM: There are only animal experiments, no clinical trials yet.	The dangers of overuse of inflammatory factors, risk of immune overactivation and then infection, cancer (201–203)
	Anti-CD8 antibody reduces CD8^+^ T cells (204)	Acute leukemia; Promising use in T2DM	Acute leukemia: Anti-CD8 antibodies are in early-phase clinical trials for the treatment of acute leukemia (205). T2DM: There are only animal experiments, no clinical trials yet.	High preparation cost, cumbersome production, risk of immunosuppression (206)
Regulate inflammatory cytokines	HYWD reduces pro-inflammatory factors (207)	Metabolic syndrome; T2DM	Metabolic syndrome: Under clinical trials with metabolic syndrome (208). T2DM: It has long been used to treat T2DM in clinical practice (209).	Generally safe, possible herb-drug interactions, requires standardization
	Subcutaneous injection of TNF-α-inhibiting adalimumab (211)	Pediatric conditions(rheumatology, gastroenterology and dermatology); Promising treatment for T2DM	Pediatric conditions: Approved in 2008 for a variety of pediatric conditions (210). T2DM: There are only animal experiments, no clinical trials yet.	Across different indications, serious adverse events such as infection, nasopharyngitis, and headache (212)
	Overexpression of anti-inflammatory factor IL-10 (213)	Hematological disorders(B-ALL); Tumors; Potential treatment for T2DM	B-ALL and Tumors: promising efficacy in in clinical trials against relapsed/refractory B-ALL (215, 216). T2DM: There are only animal experiments, no clinical trials yet.	limited therapeutic efficacy of recombinant IL-10, risk of immunosuppression (214, 215)
	Sitagliptin reduces IL-1β (217)	Widely used in clinical including T2DM	T2DM: favorable efficacy in clinical trials for the treatment of T2DM (218–221).	Generally safe, short-term headaches (222)
	Targeting the high glucose/Hif-1α transcriptional axis limits Th1 secretion of IFN-γ (223)	Potential treatment for T2DM complication	While an inverse association of the HIF-1α Pro582Ser polymorphism with diabetic nephropathy risk has been identified in genetic studies of patients and mouse models, its translation into clinical and therapeutic research is not yet available (224).	Unclear
Macrophage polarization	IL-17 blocking antibody inhibits M1 polarization (225)	Autoimmune diseases (psoriasis, psoriatic arthritis, and ankylosing spondylitis) Potential treatment for T2DM	Autoimmune diseases: the treatment remains in early-stage clinical trials (226). T2DM: There are only animal experiments, no clinical trials yet.	Mental disorders, infections, nasopharyngitis, and injection site reactions (227)
	Injection of IL-25 promotes M2 polarization (228)	Potential to treat T2DM	T2DM: There are only animal experiments, no clinical trials yet.	Immune regulatory disorders (230)
	Trisulfide-bonded lipid nanoparticles induce M2 polarization (231)	infectious diseases, cancers, and genetic disorders; Promising treatment for T2DM	Infectious diseases, cancers, and genetic disorders: Lipid nanoparticles have entered the clinical stage for treatment (232). T2DM: There are only animal experiments, no clinical trials yet.	Risks include nanoparticle accumulation (232)
	cLPT@siRNA nanocomposite to promote M2 polarization (233)	Promising treatment for T2DM	T2DM: There are only animal experiments, no clinical trials yet.	Unclear
	Injection of mesenchymal stem cells (MSCs) induces M2 polarization (234)	Pulmonary dysfunction, neurological diseases, metabolic diseases, skin burns, cardiovascular diseases, T1DM, T2DM	The treatment of various human diseases has entered the clinical stage (235–237).	Donor heterogeneity, long-term safety concerns, lack of standardized protocols and high production costs (236)

## 6 Conclusion

**Figure 4:**
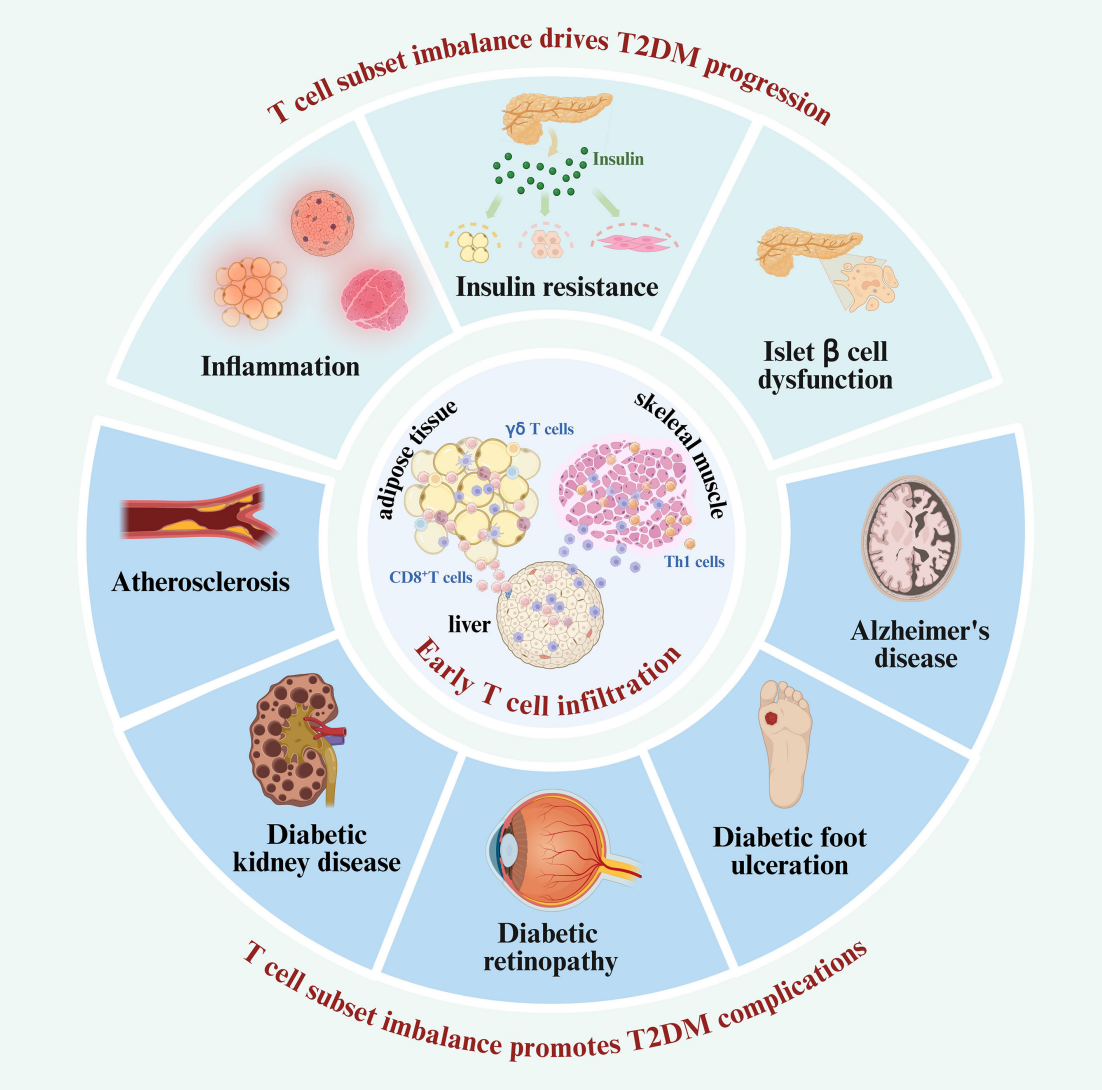
T cell subset imbalance participates in the T2DM and its complications. The critical role of T cell subset imbalance in the progression and complications of T2DM. T cells infiltrate insulin-sensitive tissues early in the disease course. Then, the imbalance of T cell subsets potentially drives T2DM progression by initiating inflammation, impairing insulin sensitivity, and inducing islet β-cell dysfunction. As T2DM advances, the dysregulated T cell subsets continued infiltration extends beyond insulin-responsive tissues, contributing to the development of various diabetic complications, including AS, DKD, DR, and other diabetes-associated conditions.

In this review, we examine the pivotal role of T cell subset imbalance in the pathological processes of T2DM and its associated complications. T cells are among the first immune cells to infiltrate insulin-responsive tissues. Then, an increase in pro-inflammatory subsets including CD8^+^ T cells, Th1, Th17, and Th22 cells, alongside a reduction in anti-inflammatory subsets such as Tregs,Th2 cells and γδT cells, leads to an imbalance which initiates inflammation. This imbalance of T cell subsets promotes the recruitment and polarization of M1 macrophages and elevates pro-inflammatory cytokine production, thereby inducing insulin resistance, advancing the progression of T2DM.Additionally, T cell subset imbalance contributes to islet inflammation and β-cell dysfunction. These findings support the new notion that the imbalance of T cell subsets is a central driver of T2DM progression and offer valuable insights into disease pathogenesis. As T2DM progresses, T cells exhibit a pro-inflammatory shift even in non–insulin-sensitive tissues, such as cardiovascular system, kidneys, retina, brain, and peripheral tissues. The imbalance in T cell subsets promotes the occurrence and development of T2DM complications primarily by inducing tissue inflammation, contributing to conditions such as AS, DKD, DR, AD, and DFU (**Figure 4**). Given the involvement of T cell subsets in T2DM and its complications, this review also discusses immunotherapeutic strategies that target T cell dysregulation—including restoring T cell subset balance, modulating pro- and anti-inflammatory cytokines derived from T cells, and altering macrophage polarization induced by pro-inflammatory T cells. Although this review underscores the critical role of T cell subset imbalance in the pathogenesis and treatment of T2DM and its complications, the specific reasons and network mechanisms driving this imbalance remain elusive. Furthermore, T2DM immunotherapy still faces certain clinical application limitations and potential safety risks, necessitating further clinical exploration.

## Glossary

T2DM: Type 2 diabetes mellitus
Th: T helper
Tregs: regulatory T cells
AS: atherosclerosis
DKD: diabetic kidney disease
DR: diabetic retinopathy
DCs: dendritic cells
HFD: high fat diet
NETs: neutrophil extracellular traps
IL: Interleukin
IFN-γ: interferon-γ
ROS: reactive oxygen species
MHC: major histocompatibility complex
CD40L: CD40 ligand
TNF-α: tumor necrosis factor-α
GM-CSF: granulocyte monocyte-colony stimulating factor
NAFLD: non-alcoholic fatty liver disease
TGF-β: transforming growth factor-beta
GLUT4: glucose transporter type 4
IRS: insulin receptor substrates
STAT: signal transducers and activators of transcription
JAK: Janus kinase
SOCS: suppressor of cytokine signaling
PI3K: phosphoinositide 3-kinase
MAPK: mitogen-activated protein kinase
JNK: c-Jun N-terminal kinase
IKK: inhibitor of kappa B kinase
PTEN: phosphatase and tensin homolog
TYK2: tyrosine kinase 2
iNKT: invariant natural killer T
CCL: C-C chemokine motif ligand
sFGL2: soluble fibrinogen-like protein 2
NF-κB: nuclear factor kappa-B
DP: diabetes-prone
MMPs: matrix metalloproteinases
VCAM-1: vascular cell adhesion molecule-1
ICAM-1: intercellular adhesion molecule-1
APOL1: Apolipoprotein L1
MCP-1: Monocyte Chemoattractant Protein-1
RMCs: retinal Müller cells
VEGF: vascular endothelial growth factor
Prf: perforin
GzmA/B: granzyme A/B
DFU: diabetic foot ulceration
AD: Alzheimer’s disease
CNS: central nervous system
BBR: berberine
HYWD: Huayuwendan
CD: Crohn’s disease
B-ALL: B-cell acute lymphoblastic leukemia
MSCs: mesenchymal stem cells.
